# Human Milk and Lactation

**DOI:** 10.3390/nu12040899

**Published:** 2020-03-26

**Authors:** Maria Lorella Gianni, Daniela Morniroli, Maria Enrica Bettinelli, Fabio Mosca

**Affiliations:** 1 Fondazione IRCCS Ca’ Granda Ospedale Maggiore Policlinico, NICU, Via Commenda 12, 20122 Milan, Italy; daniela.morniroli@gmail.com (D.M.); fabio.mosca@unimi.it (F.M.); 2 Department of Clinical Science and Community Health, University of Milan, Via Commenda 19, 20122 Milan, Italy; mebettinelli@gmail.com

Human milk is uniquely tailored to meet infants’ specific nutritional requirements [1]. However, it is more than just “milk” since it has emerged as an evolutionary strategy to promote human well-being [2]. This dynamic and bioactive fluid allows mother–infant signalling over lactation, guiding the infant in the developmental and physiological processes. Human milk exerts protection and life-long biological effects, playing a crucial role in promoting healthy growth and optimal cognitive development [3,4]. For this evidence, the promotion of breastfeeding initiation and duration becomes paramount in all healthcare settings [5]. The latest scientific advances have provided insight into different components of human milk and their dynamic and flexible changes over time in response to several biological and environmental triggers. However, the complexity of human milk composition and the synergistic mechanisms responsible for its beneficial health effects have not yet been unravelled [4]. This special issue has brought together a variety of articles, including original works and literature reviews, further exploring the complexity of the human milk biofluid and the mechanisms underlying the beneficial effects associated with breastfeeding. In this issue, the mounting amount of data regarding human milk proteome and metabolome, gathered using advanced technological achievements such as “omics” techniques, has been reviewed, describing the multitude of bioactive components and their relationship with infants’ cognitive development, growth and immune functions [6,7]. Changes in human milk protein content over the first months of lactation in mothers from different geographical and ethnic origins have been investigated [8]. The high abundance of immune active proteins reflects the well-known immunological properties of mothers’ milk [8]. Authors enhance the importance of passive immunisation through mothers’ antibodies transfer from breast milk, which has a key role for infant immune protection in the first months [9]. Differences in oligosaccharides content between term and preterm milk have also been examined in view of the potential implications for preterm infants’ clinical outcomes with special regard to their increased vulnerability to infections [10]. Given the widely known anti-inflammatory and antimicrobial properties of human milk, authors have also explored its implementation as a powerful therapeutic agent for skin issues, suggesting its potential use in settings with limited access to medicine [11]. 

In this special edition, attention has been focused on the variability of human milk compounds depending on individual differences among mothers and, far more significant, on mothers’ nutritional status and anthropometric characteristics. Authors outline the importance of a healthy lifestyle and a correct micro and macronutrient intake, before and during pregnancy and lactation, in order to promote adequate levels of vitamins and other components in human milk [12,13,14,15,16]. Moreover, author recommendations indicate the need for identifying women at risk for a deficiency, who could, therefore, benefit from an appropriate supplementation aimed at increasing breastmilk micronutrient content [12,13,14,15,16]. 

The more the exceptional qualities of human milk are brought up, the more the support of breastfeeding initiation and duration becomes fundamental [5]. However, breastfeeding rates are still lower than recommended, especially in developed countries. Authors highlight the association among breastfeeding difficulties in the first months of lactation and early breastfeeding cessation and advocate the provision of continued tailored breastfeeding support also after hospital discharge [17]. Within this context, the effectiveness of online sources including an expert instructional video in improving maternal knowledge and confidence regarding antenatal colostrum expressing, which in turn may promote long term breastfeeding, has been explored [18]. 

In this issue, authors have investigated the potential relationship between the presence of unique components of human milk and the positive long-life beneficial effects associated with breastfeeding. In view of the crucial role of neuronic acid in white matter development, its content in human milk through the first month of lactation has been quantified and compared with that of formula milk from three fat sources [19]. Human milk’s hormonal content, which seems to be involved in infants’ metabolic pathways, including appetite and energy balance, has been also examined in light of the reduced risk of developing overweight and metabolic syndrome in human milk-fed infants [20,21]. 

Benefits of human milk feeding are indeed even more critical among specific populations at high risk of developing adverse outcomes, as preterm infants [22]. This value is highlighted not only by the positive effects that human milk has in modulating preterms’ outcomes at every level but also by the results of studies in this issue demonstrating the higher levels of bioactive, micro and macronutrient contents in preterm milk, compared to full-term [10,23,24,25,26]. Within this context, however, authors have underlined the potential lack of mineral content of preterm milk that should be taken into consideration in the approach to the fortification of milk for the preterm population [27].

Since human milk feeding is associated with several life-long important beneficial health effects, in a dose-dependent relation, its promotion and support should be considered as a public health issue [2]. Unfortunately, the authors underline that breastfeeding initiation and duration are even more challenging in preterm infants [28]. Therefore, donor human milk has been studied for its role as a fresh mother’s milk substitute. Even though donor milk has to be processed through pasteurisation for microbiological safety reasons and supplemented with fortifiers, it has been demonstrated to be a better feeding alternative for preterm infants, compared to formula milk, when the own mother’s milk is not available [29]. The refrigeration, freezing, and pasteurisation of donor milk have a variable impact on vitamin, enzymes and nutrients concentration, resulting in a diminished bioactive function of donor milk [30]. In this issue, changes in concentrations after pasteurization of water-soluble forms of choline, which is crucial for infants’ development, have been investigated together with the potential for reducing the loss of donor human milk compounds by using innovative techniques including high-pressure processing [31,32].

As the diverse articles in this special issue highlight, commitment towards filling the knowledge gap on the complex and highly dynamic human milk composition and the strictly interrelated mechanisms underpinning its positive long-life biological effects is crucial for a deeper understanding of the biology of the developing infant and the optimisation of infant feeding, particularly that of the most vulnerable infants.
